# Supplementary material for: Adventitial fibroblasts direct smooth muscle cell-state transition in pulmonary vascular disease
Source: eLife. 2025 Apr 10;13:RP98558. doi: 10.7554/eLife.98558 (PMC11984959; doi:10.7554/eLife.98558)
Supplement: Supplementary file 1. — Age and sex of healthy controls (donors) and patients with pulmonary vascular disease (IPAH) with corresponding clinical data (mean pulmonary arterial pressure, mPAP, cardiac output) and PAH therapy. [file elife-98558-supp1.docx]

| **Sample** | **Sex** | **Age at transplant** | **Application** | **mPAP (mmHg)** | **Cardiac output (L/min)** | **PAH therapy** |
| --- | --- | --- | --- | --- | --- | --- |
| donor 1 | male | 45 | omics, cellular assays, GAG quantification |  |  |  |
| donor 2 | female | 58 | omics, cellular assays, GAG quantification |  |  |  |
| donor 3 | female | 44 | omics, GAG quantification, cellular assays |  |  |  |
| donor 4 | male | 23 | omics, GAG quantification, cellular assays |  |  |  |
| donor 5 | female | 24 | GAG quantification, cellular assays |  |  |  |
| donor 6 | female | 40 | GAG quantification, cellular assays |  |  |  |
| donor 7 | male | 62 | GAG quantification, stainings, cellular assays |  |  |  |
| donor 8 | male | 53 | GAG quantification, stainings, cellular assays |  |  |  |
| donor 9 | female | 56 | GAG quantification, stainings, cellular assays |  |  |  |
| donor 10 | female | 76 | GAG quantification, stainings, cellular assays |  |  |  |
| IPAH 1 | female | 32 | omics, GAG quantification, cellular assays | 50 | n.a. | bosentan, sildenafil, epoprostenol |
| IPAH 2 | female | 38 | omics, cellular assays, GAG quantification | 76 | 1.9 | bosentan, sildenafil, treprostinil |
| IPAH 3 | male | 36 | omics, cellular assays, GAG quantification | 62 | 4.3 | macitentan, sildenafil, treprostinil |
| IPAH 4 | male | 42 | omics, GAG quantification, cellular assays | 101 | 3.39 | bosentan, sildenafil, treprostinil |
| IPAH 5 | female | 27 | GAG quantification, cellular assays | 65 | 3.7 | sildenafil, treprostinil |
| IPAH 6 | female | 52 | GAG quantification, cellular assays | 63 | 2.8 | bosentan, treprostinil |
| IPAH 7 | male | 21 | GAG quantification, stainings, cellular assays | 90 | 3.1 | bosentan, sildenafil |
| IPAH 8 | male | 37 | GAG quantification, stainings, cellular assays | 68 | 5.4 | macitentan, riociguat, treprostinil |
| IPAH 9 | female | 52 | GAG quantification, stainings, cellular assays | 77 | n.a. | bosentan, sildenafil, treprostinil |
| IPAH 10 | female | 32 | GAG quantification, stainings, cellular assays | 74 | n.a. | macitentan, riociguat, treprostinil |
