# Supplementary material for: Adventitial fibroblasts direct smooth muscle cell-state transition in pulmonary vascular disease
Source: eLife. 2025 Apr 10;13:RP98558. doi: 10.7554/eLife.98558 (PMC11984959; doi:10.7554/eLife.98558)
Supplement: Supplementary file 2. [file elife-98558-supp2.docx]

| **Antibody** | **Company** | **Catalog number** | **Dilution** | **Detection** | **ID** |
| --- | --- | --- | --- | --- | --- |
| ACTA2-Cy3 | Sigma | C6198 | 1:100 | primary labeled Ab | AB_476856 |
| ACTA2-FITC | Sigma | F3777 | 1:100 | primary labeled Ab | AB_476977 |
| ADH1A/ADH1C | Atlas Antibodies | HPA047814 | 1:300 | Tyramide signal amplification | AB_2680163 |
| AFAP1 | Atlas Antibodies | HPA015642 | 1:200 | Tyramide signal amplification | AB_1844632 |
| CD45 | Abcam | ab10558 | 1:300 | Tyramide signal amplification | AB_442810 |
| CSRP1 | Atlas Antibodies | HPA045617 | 1:100 | Tyramide signal amplification | AB_2679391 |
| ITGA2 | Atlas Antibodies | HPA063556 | 1:100 | Tyramide signal amplification | AB_2685040 |
| MGST1 | ThermoFisher Scientific | PA5-60845 | 1:100 | Tyramide signal amplification | AB_2643943 |
| PCNA | Santa Cruz | sc-7907 | 1:100 | secondary labeled Ab | AB_2160375 |
| PDGFRalpha | Cell Signaling Technologies | 3174 | 1:50 | Tyramide signal amplification | AB_2162345 |
| PDGFRalpha | Abcam | ab203491 | 1:500 | Tyramide signal amplification | AB_2892065 |
| RGS5 | Santa Cruz | sc-514184 | 1:2000 | Tyramide signal amplification | - |
| TMX2 | Atlas Antibodies | HPA063763 | 1:100 | Tyramide signal amplification | AB_2685116 |

| **Secondary detection reagents** | **Company** | **Catalog number** | **Dilution** |
| --- | --- | --- | --- |
| Donkey anti-Rabbit IgG AF-555 | Thermo Fisher Scientific | A-31572 | 1:400 |
| Immpress horse anti- mouse HRP-polymer | Vector Labs | MP-7402 | RTU |
| Immpress horse anti- rabbit HRP-polymer | Vector Labs | MP-7401 | RTU |
| CF405L tyramide dye | Biotium | 92198 | 1:200 |
| CF620R tyramide dye | Biotium | 92194 | 1:200 |
| Opal 7-Color Manual IHC Kit | Akoya Biosciences | SKU NEL811001KT | RTU |
| CF594 TUNEL assay kit | Biotium | 30064 | - |
| DAPI | Thermo Fisher Scientific | 62248 | 1:1000 |
