# Supplementary material for: Adventitial fibroblasts direct smooth muscle cell-state transition in pulmonary vascular disease
Source: eLife. 2025 Apr 10;13:RP98558. doi: 10.7554/eLife.98558 (PMC11984959; doi:10.7554/eLife.98558)
Supplement: Supplementary file 3. [file elife-98558-supp3.docx]

| species | gene symbol |  | primer sequence (5´ to 3´) |
| --- | --- | --- | --- |
| human | MYH11 | forward | TTGGCTCCCACGATGTAACC |
|  |  | reverse | AAGCAGCTTCTACAAGCAAAC |
| human | RHOA | forward | AGCAAGCATGTCTTTCCACA |
|  |  | reverse | GAAGAGGCTGGACTCGGATT |
| human | BGN | forward | CTGGCATCCCCAAAGACCTC |
|  |  | reverse | CCAGTTCGATGGCCTGGATT |
| human | VCAN | forward | TGTTAATCGTGTGGGCCATGA |
|  |  | reverse | AGAAGCTGTCTGGCTGGTTG |
| human | CNN1 | forward | CCCCACGACATTTTTGAGGC |
|  |  | reverse | CACTCCCACGTTCACCTTGT |
| human | PPARG | forward | AGAGCCTTCCAACTCCCTCA |
|  |  | reverse | TCCGGAAGAAACCCTTGCAT |
| human | TNFRSF11B | forward | CAGTGTCTTTGGTCTCCTGC |
|  |  | reverse | TCCTCACACAGGGTAACATCTATT |
| human | CCND1 | forward | AGTGGAAACCATCCGCCG |
|  |  | reverse | TCTGTTCCTCGCAGACCTCCA |
| human | DCN | forward | GCATTCCTCAAGGTCTTCCTCC |
|  |  | reverse | AGCCATTGTCAACAGCAGAG |
| human | B2M | forward | CCTGGAGGCTATCCAGCGTACTCC |
|  |  | reverse | TGTCGGATGGATGAAACCCAGACA |
| human | HMBS | forward | CTGCAACGGCGGAAGAAAA |
|  |  | reverse | AATCTTGTCCCCTGTGGTGG |
